# Head-To-Head Comparison of Treatment Failure and Costs among COPD Patients Who Used Noninvasive Ventilation in the Ward versus in the ICU: A Propensity-Matched Cohort Study

**DOI:** 10.1155/2020/6682589

**Published:** 2020-12-31

**Authors:** Yueling Hong, Qiao Liu, Linfu Bai, Lei Jiang, Xiaoli Han, Shicong Huang, Wenhui Hu, Jun Duan, Chuanbo Liu

**Affiliations:** ^1^Department of Respiratory and Critical Care Medicine, The First Affiliated Hospital of Chongqing Medical University, Chongqing 400016, China; ^2^Department of Critical Care Medicine, The People's Hospital of Gaoxin District, Chongqing 400039, China

## Abstract

**Background:**

Head-to-head comparison of treatment failure and costs among chronic obstruct pulmonary disease (COPD) patients who used noninvasive ventilation (NIV) in the ward versus in the ICU is lacking.

**Methods:**

This retrospective study was performed in a department of respiratory and critical care medicine in a teaching hospital. COPD patients who used NIV in the respiratory ward or respiratory ICU were screened. We enrolled patients with PaCO_2_ more than 45 mmHg and pH less than 7.35 before the use of NIV.

**Results:**

We enrolled 83 patients who initiated NIV in the ward and 319 patients in the ICU. Only 5 (6%) patients in the ward were required to transfer to ICU for intensive care. The vital signs were worse but improved faster within 24 h of NIV among patients in the ICU than those in the ward. The NIV failure, hospital mortality, and the length of stay in hospital did not differ between the two groups. However, the duration of NIV was shorter (median 4.0 vs. 6.1 days, *p* < 0.01) and hospital costs were higher (median 4638 vs. 3093 $USD, *p* < 0.01) among patients in the ICU than those in the ward. After propensity matching, 42 patients were left in each group, and the baseline data were comparable between the two groups. The findings in the overall cohort were confirmed again in the propensity-matched cohort.

**Conclusions:**

Among COPD patients, the use of NIV in the ward leads to longer duration of NIV, but lower hospital costs, and similar NIV failure and mortality compared with those in the ICU.

## 1. Introduction

The prevalence of chronic obstructive pulmonary disease (COPD) is 8.6% in adult population and 13.7% in those aged 40 years or older [1]. It leads to high morbidity and mortality and becomes the third leading cause of death in China [2, 3]. Also, the cost burden in COPD patients is much higher than non-COPD subjects [4]. At the end stage, shortness of breath and hypercapnia are the main clinical features in COPD patients. Therefore, relief of dyspnea and reduction of PaCO_2_ are two main therapeutic regimens.

In COPD patients, noninvasive ventilation (NIV) improves minute ventilation and decreases the work of breathing [5]. NIV also reduces the need of intubation and mortality in this population [6, 7]. Guidelines have strongly recommended the use of NIV in hospitalized patients due to COPD exacerbations [8–10]. Also, the use of NIV in hospitalized COPD patients is increasing year by year [11].

Among COPD patients, the use of NIV in the intensive care unit (ICU) is common [12–14]. As ICU beds are limited, not all COPD patients can admit to ICU for application of NIV. The ward is another place where the NIV can be used. Several studies have reported that use of NIV in the ward is feasible [15–17]. However, head-to-head comparison of treatment failure and costs among COPD patients who used noninvasive ventilation (NIV) in the ward versus in the ICU is lacking. Here, we aimed to explore the optimal location to use of NIV in COPD patients.

## 2. Methods

This was a retrospective observational study performed in a teaching hospital from March, 2015, to June, 2018. We screened all the patients who used NIV as first-line therapy due to COPD exacerbation in the respiratory ward or respiratory ICU and enrolled the patients with pH less than 7.35 and PaCO_2_ more than 45 mmHg. However, the patients with refusal of intubation were excluded. The diagnosis of COPD was based on the criteria published by Chinese Medical Association [18]. The study protocol was approved by our ethics committee (the First Affiliated Hospital of Chongqing Medical University). As this was a retrospective study, the informed consent was waived.

In the respiratory ward, the bed nurse ratio was 1 : 0.4 and the bed physician ratio was 1 : 0.5. In the respiratory ICU, the bed nurse ratio was 1 : 2.5 and the bed physician ratio was 1 : 0.6. Use of NIV in the ward or in the ICU was decided by the attending physicians based on the volume of ICU beds, ability to pay, and patients' wishes. In our hospital, the indications of NIV in patients with COPD exacerbation were as follows: (1) respiratory rate more than 25 breaths/min, (2) PaCO_2_ more than 45 mmHg, (3) pH less than 7.35, (4) PaO_2_/FiO_2_ less than 200 mmHg, and (5) vigorous activity of accessory respiratory muscles [19].

The pneumologist took care of the NIV patients in the ward. When the NIV has been used for more than 2 hours, the patients in the ward were considered to be transferred to ICU for escalation therapy if the respiratory failure got worse. The criteria were respiratory rate more than 35 breaths/min, pH less than 7.25, PaO_2_/FiO_2_ less than 100 mmHg, unstable hemodynamic status, and other causes required intensive care. However, this was decided by the attending physician's discretion.

The management of NIV (BiPAP Vision or V60; Philips Respironics, Carlsbad, CA) in COPD patients was based on our hospital protocol [19–21]. The face mask was the first choice for the interface to connect the ventilator to the patient. The size of the face mask was fitted to the face type. Bilevel positive airway pressure was used for all patients. The expiratory positive airway pressure was initially set at 4 cm H_2_O, and it was titrated to diminish the ineffective trigger. Usually, the expiratory positive pressure ventilation was maintained at 4 to 8 cm H_2_O. The inspiratory positive airway pressure was initially set at 8 cm H_2_O and then gradually increased to achieve the best control of dyspnea or to the tolerance of the patient. Usually, the inspiratory positive airway pressure was maintained at 15 to 20 cm H_2_O within 30 minutes. The fractional concentration of inspiratory oxygen (FiO_2_) was adjusted to maintain the bedside SpO_2_ above 90% and the PaO_2_ above 60 mmHg. At the initial phase, continuous use of NIV was encouraged except drinking, eating, and sputum excretion. If the respiratory failure relieved, intermittent use of NIV was performed until the NIV was weaned. However, in those whose respiratory failure progressively deteriorated and required invasive mechanical ventilation, intubation was performed. The NIV failure was defined as requirement of intubation or death during NIV intervention. In addition, the decision to transfer the patient to ICU for escalation therapy was based on the attending physicians' discretion if the respiratory failure progressively deteriorated.

We collected the age, gender, underlying disease, vital signs, and arterial blood gas tests. The disease severity was assessed by the acute physiology and chronic health evaluation II (APACHE II) score. The prognostic outcomes were also collected including the rate of NIV failure and hospital mortality. The resource consumption was assessed including the duration of NIV, length of stay in hospital, and hospital cost. All the data we collected were extracted from the medical records.

### 2.1. Statistical Analysis

The continuous variable was reported as mean value and standard deviation or median value and interquartile range (IQR) when appropriate. Differences between two groups were analyzed with the use of Student's *t*-test or the Mann–Whitney *U* test. Categorical variable was reported as numbers and percentages, and the differences between groups were analyzed with the use of Chi-square or Fisher's exact tests. *p* values less than 0.05 were considered statistically significant.

Propensity scores were estimated using multiple logistic regression analyses, with adjustments for age, gender, underlying disease, APACHE II score, GCS, respiratory rate, heart rate, mean arterial pressure, pH, PaCO_2_, and PaO_2_/FiO_2_. After calculating propensity scores, we matched the patients who initiated the NIV in the ward and those in the ICU with similar propensity scores at a 1 : 1 ratio, using the nearest neighbor method, no replacement, and a 0.05 caliper width.

## 3. Results

In the overall cohort, 83 patients initiated NIV as first-line therapy in the ward and 319 patients in the ICU (Table 1). Compared with the subjects in the ICU, the patients who initiated the NIV in the ward had lower respiratory rate (24 ± 3 vs. 28 ± 6 breaths/min, *p* < 0.01), lower heart rate (98 ± 19 vs. 108 ± 22, beats/min), higher pH (7.29 ± 0.05 vs. 7.26 ± 0.06, *p* < 0.01, higher PaO_2_/FiO_2_ (228 ± 70 vs. 208 ± 90 mmHg, *p*=0.05), and higher Glasgow coma scale (14.8 ± 0.7 vs. 14.4 ± 1.5, *p*=0.02) at the beginning of NIV.

**Figure 1:**
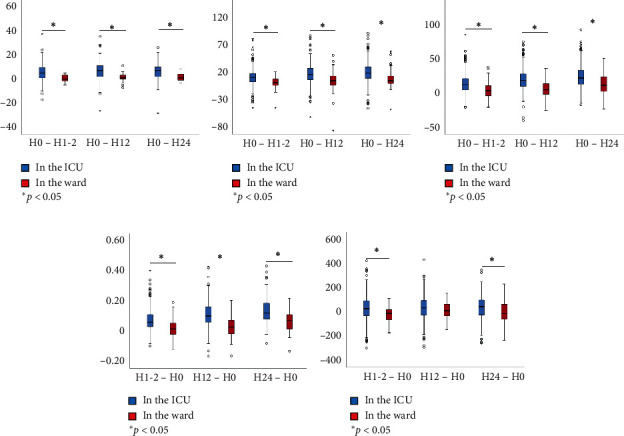
The changes of vital signs and arterial blood gas tests within 24 h of NIV in overall cohort. (a) ΔRespiratory rate, breaths/min. (b) ΔHeart rate, beats/min. (c) ΔPaCO_2_, mmHg. (d) ΔpH. (e) ΔPaO_2_/FiO_2_, mmHg.

Five (6%) patients in the ward were transferred to the ICU for intensive care (3 for continuous use of NIV and 2 for intubation) due to progressive deterioration (Table 2). Of the 5 patients, only one died in hospital. The rate of NIV failure, hospital mortality, and the length of stay in hospital did not differ between the two groups. The duration of NIV was longer in the patients who initiated NIV in the ward than those in the ICU (6.1, IQR: 3.0–9.1 vs. 4.0, 2.1–6.6 days, *p* < 0.01). But, their hospital cost was much lower (3093, IQR: 2214–4352 vs. 4638, 3259–7712 $USD, *p* < 0.01). Also, the vital signs from initiation to 24 h of NIV improved faster among the patients in the ICU than those in the ward (Figure 1).

**Figure 2:**
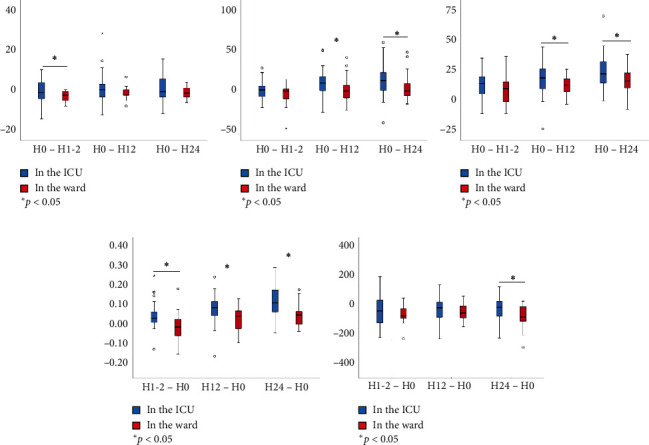
The changes of vital signs and arterial blood gas tests within 24 h of NIV in propensity-matched cohort. (a) ΔRespiratory rate, breaths/min. (b) ΔHeart rate, beats/min. (c). ΔPaCO_2_, mmHg. (d) ΔpH. (e) ΔPaO_2_/FiO_2_, mmHg.

After propensity matching, 42 patients were left in each group (Table 3). The baseline data were comparable between the two groups. The rate of NIV failure and hospital mortality did not differ between the two groups (Table 4). Similar with the overall cohort, we also found that the duration of NIV was longer and hospital cost was lower in patients who initiated the NIV in the ward than those in the ICU. Also, the respiratory rate, heart rate, pH, PaCO_2_, and PaO_2_/FiO_2_ improved faster within the first 24 h of NIV in patients who initiated NIV in the ICU than those in the ward (Figure 2). In addition, there was another new finding that the length of stay in hospital was shorter among the patients in the ICU than those in the ward (median 8.8 vs. 10.9 day, *p*=0.04).

## 4. Discussion

To our knowledge, this is the first head-to-head comparison of treatment failure and costs among COPD patients who used NIV in the ward versus in the ICU. Slower improvement of vital signs and arterial blood gas tests, longer duration of NIV, but lower cost were observed in patients who used NIV in the ward than those in the ICU. Also, the rate of NIV failure and hospital mortality did not differ between the two groups.

A landmark paper published at 2000 has reported that the early use of NIV in the ward among patients with COPD exacerbation reduced the need for intubation and hospital mortality compared with standard care [6]. This study only demonstrated that the use of NIV in the ward was feasible. During the following 20 years, many studies also showed the benefits of NIV in the ward among COPD population [17, 22, 23]. However, these studies only demonstrated that patients with COPD exacerbation benefited from NIV in the ward but failed to demonstrate the optimal location where the NIV should be used. To our knowledge, our study is the first one head-to-head comparison on the use of NIV in the ward against those in the ICU. It provides a reference for clinical staffs how to select the location to use NIV.

We found an interesting result that patients who used NIV in the ward had a less severe respiratory acidosis and lower cost but a longer duration of NIV compared with those in the ICU. The potential reasons were as follows. First, the physicians and nurses in the ICU were much more than those in the ward when they managed the same number of patients. Second, the ICU physicians and nurses have managed much more NIV patients than those who managed NIV in the ward. So, the experience on NIV management was much richer in ICU physicians and nurses. Third, the two reasons lead to a faster improvement in vital signs and arterial blood gas tests in the ICU patients. Therefore, these reasons can be explained this interesting result.

Delayed admission to the ICU may be associated with increased mortality. A previous study reported by Valentini et al. showed that the mortality was 18% in patients who directly transferred from the emergency department to respiratory ICU, but it increased to 64% in cases who transferred from the respiratory ward [24]. This study enrolled all the patients who used NIV in the ICU due to various reasons. However, we only enrolled patients who used the NIV due to COPD exacerbation, and only 5 (6%) patients in the ward were transferred to ICU due to progressive deteriorations. Among the 5 cases, only one died in hospital. Therefore, the need to transfer to the ICU for escalation therapy is low among COPD patients who used NIV in the ward. Also, the mortality is not high among those who transferred to the ICU. We believe the use of NIV in the ward among COPD patients is an alternative place.

Our study may be limited by the retrospective design. Where to use the NIV was decided by the attending physicians. More serious illness patients were more likely to transfer to the ICU, which lead to unbalanced baseline data between patients in the ICU and those in the ward. However, we performed a propensity-matched analysis to balance the confounders. After propensity matching, the baseline data were comparable. This improves the comparability between the two groups. In addition, the transportation of the patients from the ward to ICU for escalation therapy was also decided by the attending physicians if the respiratory failure progressively deteriorated. Delayed admission to the ICU for escalation therapy may be occurred because the personnel allocation was much lower in the ward than that in the ICU. Therefore, more attention should be paid to the patients who used the NIV in the ward.

## 5. Conclusions

The use of NIV in the ward is cost effective for COPD patients. The rate of transportation to the ICU for escalation therapy is low. NIV failure rate and mortality did not differ between patients who initiated NIV in the ward and those in the ICU.

## Figures and Tables

**Table 1:** Clinical characteristics of the patients who used NIV in the ward versus those in the ICU.

	In the ward *N* = 83	In the ICU *N* = 319	*p*
Age, years	71 ± 8	73 ± 9	0.10
Male/female	63/20	243/76	>0.99
Underlying disease			
Hypotension	27 (33%)	110 (35%)	0.80
Diabetes mellitus	16 (19%)	61 (19%)	>0.99
Chronic heart disease	13 (16%)	63 (20%)	0.44
Chronic kidney disease	0 (0%)	14 (4%)	0.08
Chronic liver disease	1 (1%)	10 (3%)	0.47
Data collected at initiation of NIV			
APACHE II score	15 ± 2	16 ± 4	0.09
GCS	14.8 ± 0.7	14.4 ± 1.5	0.02
Respiratory rate, breaths/min	24 ± 3	28 ± 6	<0.01
Heart rate, beats/min	98 ± 19	108 ± 22	<0.01
Mean arterial pressure, mmHg	91 ± 11	100 ± 18	<0.01
pH	7.29 ± 0.05	7.26 ± 0.06	<0.01
PaCO_2_, mmHg	80 ± 13	83 ± 17	0.08
PaO_2_/FiO_2_, mmHg	228 ± 70	208 ± 90	0.05

NIV = noninvasive ventilation, ICU = intensive care unit, GCS = Glasgow coma scale, APACHE II = acute physiology and chronic health evaluation II.

**Table 2:** Outcomes in patients who used NIV in the ward versus those in the ICU.

	In the ward *N* = 83	In the ICU *N* = 319	*p*
NIV failure	4 (5%)	35 (11%)	0.10
Hospital mortality	3 (4%)	31 (10%)	0.08
Transfer to the ICU	5 (6%)	−	−
Duration of NIV, days	6.1 (3.0–9.1)	4.0 (2.1–6.6)	<0.01
The length of stay in hospital, days	10.2 (8.0–15.0)	10.2 (6.7–15.0)	0.40
Hospital cost, $	3093 (2214–4352)	4638 (3259–7712)	<0.01

NIV = noninvasive ventilation, ICU = intensive care unit.

**Table 3:** Clinical characteristics in the propensity-matched cohort.

	In the ward *N* = 42	In the ICU *N* = 42	*p*
Age, years	72 ± 8	72 ± 9	0.88
Male/female	32/10	33/9	>0.99
Underlying disease			
Hypotension	16 (38%)	12 (29%)	0.48
Diabetes mellitus	10 (24%)	11 (26%)	>0.99
Chronic heart disease	6 (14%)	9 (21%)	0.57
Chronic kidney disease	0 (0%)	1 (2%)	>0.99
Chronic liver disease	0 (0%)	1 (2%)	>0.99
Data collected at initiation of NIV			
APACHE II score	15 ± 3	15 ± 4	0.58
GCS	14.7 ± 0.9	14.5 ± 0.8	0.31
Respiratory rate, breaths/min	25 ± 3	26 ± 8	0.49
Heart rate, beats/min	104 ± 20	106 ± 19	0.31
Mean arterial pressure, mmHg	93 ± 12	95 ± 15	0.47
pH	7.29 ± 0.04	7.29 ± 0.05	0.69
PaCO_2_, mmHg	77 ± 11	76 ± 16	0.82
PaO_2_/FiO_2_, mmHg	208 ± 71	214 ± 92	0.73

NIV = noninvasive ventilation, ICU = intensive care unit, GCS = Glasgow coma scale, APACHE II = acute physiology and chronic health evaluation II.

**Table 4:** Outcomes in the propensity-matched cohort.

	In the ward *N* = 42	In the ICU *N* = 42	*p*
NIV failure	1 (2%)	4 (9%)	0.36
Hospital mortality	2 (5%)	2 (5%)	>0.99
Transfer to the ICU	2 (5%)	−	−
Duration of NIV, days	7.1 (4.1–10.6)	4.2 (1.8–5.7)	<0.01
The length of stay in hospital, days	10.9 (8.6–16.1)	8.8 (6.4–15.6)	0.04
Hospital cost, $	3105 (2286–4443)	3853 (2281–8049)	0.02

NIV=noninvasive ventilation, ICU=intensive care unit.

## Data Availability

The datasets analyzed during this study are available from the corresponding author upon reasonable request.

## Abbreviations

COPD: Chronic obstructive pulmonary disease
NIV: Noninvasive ventilation
GCS: Glasgow coma scale
APACHE II: Acute physiology and chronic health evaluation II
IQR: Interquartile range
ICU: Intensive care unit
